# A sham-controlled randomised trial evaluating the safety, acceptability, and efficacy of autonomic neuromodulation using transcutaneous vagal sensory stimulation in uncontrolled hypertensive patients: rationale and study design of the SCRATCH-HTN study

**DOI:** 10.3389/fcvm.2026.1693086

**Published:** 2026-04-08

**Authors:** Ajay Gupta, David Collier, James Steckelmacher, Jane Field, Olivier Zongo, Mital Patel, George Collett, Everard Mascarenhas, Andrey Gourine, Annastazia Learoyd, Alexander V. Gourine, Peter S. Sever

**Affiliations:** 1 William Harvey Research Institute, Queen Mary University of London, London, United Kingdom; 2 Barts Heart Centre, St. Bartholomew’s Hospital, Barts Health NHS Trust, London, United Kingdom; 3 Department of Clinical Pharmacology, The Royal London Hospital, Barts Health NHS Trust, London, United Kingdom; 4 National Heart & Lung Institute, Imperial College London, London, United Kingdom; 5 Afferent Medical Solutions Ltd., Cardiff, United Kingdom; 6 Centre for Cardiovascular and Metabolic Neuroscience, Neuroscience, Physiology and Pharmacology, University College London, London, United Kingdom

**Keywords:** clinical trial, hypertension, randomised control trial (RCT), transcutaneous vagal nerve stimulation (TVNS), vagal nerve stimulation (VNS), sham-controlled, autonomic

## Abstract

**Background:**

Despite the broad availability of antihypertensive drugs, approximately 40% of hypertensive patients fail to achieve the recommended blood pressure (BP) levels and may require alternative treatment(s). At present, renal denervation is the only proven non-pharmacological device-based alternative treatment available, but it is a costly, invasive, hospital-based procedure that is unlikely to be widely available. Transcutaneous autonomic neuromodulation (tAN)—if shown to be safe, acceptable, and efficacious—can offer a non-invasive, inexpensive, self-administered device-based innovative adjunct or alternative to pharmacological therapy.

**Methods:**

SCRATCH-HTN is a double-blind, sham-controlled trial, with 63 participants randomised on a 2:1 basis to receive either tAN or sham-tAN treatment. Hypertensive patients on medication were included if they had elevated systo-diastolic BPs on daytime ambulatory BP monitoring (ABPM) [systolic BP (SBP) of ≥135 and <170 mmHg and mean daytime diastolic BP (DBP) of ≥85 and <115 mmHg]. Participants were trained to self-administer tAN therapy for 30 min every day for first 14 days and then once a week for 10 weeks. The primary endpoint was change in daytime ambulatory SBP from baseline to 3 months. Secondary endpoints included change in 24-h ambulatory and office SBP and DBP, BP variability, heart rate variability, quality of life, and sleep quality from baseline to end of treatment. Other exploratory outcomes included evaluation of impact on functional exercise (6-min walk test), structural and functional changes in the heart, cognitive function, and central blood pressures. A subgroup of patients underwent detailed autonomic functional assessment at the start and end of the study.

**Conclusion:**

The SCRATCH-HTN trial is a phase 2a study testing the safety, acceptability, and potential efficacy of tAN treatment for improving blood pressure control in patients with elevated BP despite medication. It also explores the effects of tAN on sleep, exercise tolerance, heart rate variability, central BP, cardiac structure, and autonomic function. If effective, it could offer a transformative approach to hypertension management.

**Study Protocol Registration:**

Clinicaltrials.gov, identifier NCT05179343 and ISRCTN (14509154).

## Introduction

Hypertension or high blood pressure (BP) affects more than 1.3 billion people worldwide (1). It is the leading risk factor for premature death and disability and a key contributor to global disease burden (2–4). This is driven by its role as the leading global cause of cardiovascular and cerebrovascular disease mortality and morbidity, as well as its significant contribution to the global burden of chronic kidney disease (5–7). In England, one in three adults has hypertension, rising to over 60% amongst adults aged ≥65 years (8, 9). As such, the cost to healthcare systems is significant: Hypertension is estimated to cost the UK National Health Service (NHS) more than £2.1 bn annually (10).

Despite the widespread availability of antihypertensive drugs, 40% of all patients with hypertension fail to achieve the National Institute for Health and Care Excellence (NICE) recommended BP levels (<140 mmHg systolic and <90 mmHg diastolic) (9, 10). These patients are classified as having uncontrolled hypertension. Non-adherence to prescribed medications is a major challenge, partly due to drug intolerance/adverse side effects. The global prevalence of non-adherence to antihypertensive medication in diagnosed hypertensive patients is estimated to be around 27%–40% (11) and is present in approximately 84% of those with uncontrolled hypertension (12) and 37% of those with drug-resistant hypertension (11, 13). As such, there is an urgent, unmet clinical need for an effective therapy for uncontrolled and drug-resistant hypertension.

One possible therapy is device-based autonomic neuromodulation. During the development of hypertension, parasympathetic (vagal) activity declines, while sympathetic activation increases, which can, if left untreated, lead to chronically altered homeostatic autonomic imbalance. Redressing autonomic imbalance has already been achieved using catheter-based renal denervation (14, 15), which appears to be a safe treatment for reducing arterial blood pressure (14, 16). A recent meta-analysis found modest but significant reductions in 24-h ambulatory systolic BP (SBP, mean reduction = −2.23 mmHg) and 24-h ambulatory diastolic BP (DBP, mean reduction = −1.16 mmHg) following renal denervation amongst uncontrolled hypertensive patients on antihypertensive medication (17). However, renal denervation is an invasive procedure, requiring hospitalisation and tertiary care delivered by experts (15). This limits universal adoption, while uncertainty remains regarding the duration of the blood pressure-lowering effect and the requirement for repeat procedures.

Autonomic balance can potentially be reinstated non-invasively using a method called transcutaneous autonomic neuromodulation (tAN), otherwise known as transcutaneous vagus nerve stimulation (tVNS) (18). In this method, electrical stimulation is applied to the regions of the outer ear that are innervated by sensory (afferent) fibres of several cranial and spinal nerves, including the auricular branch of the vagus nerve (18, 19). These afferent nerve fibres, constituting 80% of the vagus nerve, transmit sensory information, predominantly from visceral organs, to the nucleus tractus solitarius (NTS) where 95% of vagal afferent fibres terminate. Efferent vagal nerve fibres leaving the NTS via the dorsal vagal nucleus and nucleus ambiguous have various visceral organ targets and influence an array of homeostatic physiological functions including BP. The firing of efferent vagal nerve fibres reduces BP through a range of targets and mechanisms, including cardiovagal innervation of the heart, which produces negative inotropic and chronotropic effects, and attenuation of sympathetic activity (via inhibition of the rostroventrolateral medulla) that reduces vasoconstriction and the activity of the renin–angiotensin–aldosterone system (20–22). Recruitment of these afferent projections through transcutaneous electrical stimulation of the auricular nerve modulates the autonomic control circuits in the brainstem and acutely shifts the autonomic balance towards a net vagal dominance (23–26), as assessed by baroreflex sensitivity (27) and heart rate variability measures (19, 23, 28). These effects are consistent with vagal recruitment and sympathetic inhibition as demonstrated by beneficial effects of tAN in patients with epilepsy (29, 30), coronary artery disease (31), and atrial fibrillation (32, 33).

Recent evidence suggests that autonomic neuromodulation via unilateral tAN applied daily for 3 months reduced BP in untreated young individuals with grade-1 hypertension (34). The study was open-labelled, unblinded, and BP was measured by patients at home with significant self-reported reductions reported after 1 month. These data, however, are at odds with the results of the study by Stavrakis et al. (35) who used an identical device and treatment protocol involving unilateral daily tAN and observed no change in office BP in patients with heart failure with preserved ejection fraction; 96% of these patients were hypertensive. Most published studies, including the two referenced, that involved longer periods of tAN employed unilateral auricular stimulations, were open-labelled, and used inconsistent methods of BP monitoring, potentially leading to ascertainment bias.

The SCRATCH-HTN study (Sham-Controlled Randomised Trial Evaluating the Safety, Acceptability, and Efficacy of Autonomic Neuromodulation using Transcutaneous Vagal Sensory Stimulation in Uncontrolled Hypertensive Patients) tests the hypothesis that tAN treatment is safe and acceptable, improves the control of BP in hypertension, and improves wellbeing amongst those receiving the active treatment compared to those on sham treatment. In this study, tAN is applied to both ears (bilaterally) using a modified and improved protocol. This stimulation protocol was developed based on significant evidence indicating that applying sensory stimuli bilaterally is more effective than unilateral stimulation in inducing brain plasticity and neuromodulation (36, 37). Using this protocol in our unpublished proof-of-concept study of 10 patients with drug-resistant hypertension, bilateral tAN reduced 24-h SBP by an average of 14 mmHg at 1 month after the course of tAN compared to before the treatment (Supplementary Figure 1). This set the premise for the current trial protocol, although the findings require critical interpretation given the observational, single-arm design of the proof-of-concept study conducted in a small number of self-selected individuals. Further details on the study background and trial design rationale can be found in the clinical investigational plan (Supplementary Material 4).

The data obtained will be used to develop larger efficacy and cost-effectiveness follow-up studies. The SCRATCH-HTN trial also includes a sub-study of participants to evaluate autonomic function at baseline and at the end of the treatment period. This will provide mechanistic insights into the effects of treatment on components of the autonomic nervous system responses. The present manuscript outlines the protocol for this trial.

## Methods and analysis

### Patient and public involvement

During study development and prior to the start of the trial, a group of hypertensive patients were invited to provide feedback. Patient and public involvement representatives also served on our trial steering committee, contributing to the study design, device logbooks, and questionnaires, and overseeing the conduct of the study.

### Trial design

The SCRATCH-HTN trial is a double-blind, sham-controlled study, with block randomised on a 2:1 basis, where participants receive either tAN or sham-tAN treatment. tAN was applied using the AffeX-CT electronic device developed by Afferent Medical Solutions, Ltd (UK).

### Study population

Study participants were recruited from six participants-identifying centres (PICs) in and around London (Supplementary Table 1). For consistency, all participants were processed at a single site: the William Harvey Clinical Research Centre, Queen Mary University of London. Hypertensive patients, who were receiving between one and four antihypertensive medications, were eligible for recruitment if they had elevated systo-diastolic BP on daytime ambulatory BP monitoring ((ABPM): daytime average SBP of ≥135 and <170 mmHg, and daytime average DBP of ≥85 and <115 mmHg) and the presence of one or more of the following conditions: obesity, type 2 diabetes, heart rate ≥70 beats per minute, metabolic syndrome, dyslipidaemia, or polycystic ovarian syndrome (see Table 1 for summary of key inclusion criteria and further details in Supplementary Table 2). Exclusion criteria included atrial fibrillation, eGFR < 45 mL/min, type 1 diabetes mellitus, poorly controlled type 2 diabetes (HbA1c > 69 mmol/mol) and/or or insulin therapy, and orthostatic hypotension (defined as a fall >20 mmHg in SBP on moving from sitting to standing). The full list of exclusion criteria can be found in Supplementary Table 3.

**Table 1:** Summary of participant inclusion criteria.

Inclusion criteria
Aged ≥18 and <80 years
Taking between 1 and 4 antihypertensive medications (inclusive)
Confirmed diagnosis of hypertension
24-hour ABPM mean daytime^a^ SBP ≥ 135 mmHg and <170 mmHg and mean daytime DBP ≥85 and <115 mmHg
Moreover, participant must have **one or more** of the following associated conditions:
Obesity [Body Mass Index (BMI) > 30 kg/m^2^ or waist circumference >94 cm (men) or >80 cm (women)] NB. For participants of Southeast Asian/Chinese/Japanese origin these cut-offs are >90 cm (men) or >80 cm (women)
Type 2 diabetes-controlled or sub-optimally controlled (HbA1c ≤ 8.5% or ≤69 mmol/mol) on diet and/or medications except insulin
Heart rate (any one of the three recordings) ≥70 bpm (measurement taken after 5 min of rest in a seated position) or heart rate ≥60 bpm if taking beta-blocker medication
HbA1c ≥ 42 mmol/mol or fasting glucose (if available) ≥5.6 mmol/L **AND** either low HDL cholesterol (≤1.03 mmol/L for men and ≤1.29 mmol/L for women) or high triglycerides (≥1.7 mmol/L)
Both low HDL cholesterol (≤1.03 mmol/L for men and ≤1.29 mmol/L for women) **AND** high triglycerides (≥1.7 mmol/L)
Diagnosed or known polycystic ovarian syndrome

Full inclusion and exclusion criteria detailed in Supplementary Tables S1, S2.

^a^ Daytime defined as hours from 0700 to 2300.

### Randomisation

Eligible participants were randomised in a 2:1 ratio to the tAN and sham-tAN intervention arms, respectively, using the online randomisation tool “Sealed Envelope.” This tool employed block randomisation and a dynamic minimisation approach to ensure balanced allocation between treatment groups (38). The minimisation algorithm incorporated the following baseline factors: age (<65 or ≥65 years), sex, body mass index (BMI; <30 or ≥30 kg/m^2^), and mean daytime average SBP (<160 or ≥160 mmHg).

### Description of intervention

All randomised participants were provided with identical devices, with allocation to either the tAN or sham-tAN arm concealed. On the day of randomisation, all participants received standardised training using a dedicated training device before being assigned their personal study device. Each participant was provided with an individually set stimulation threshold as well as access to further training resources via a user guide (see Supplementary Material 2—User Guide), demonstration video, and telephone support.

**Figure 1:**
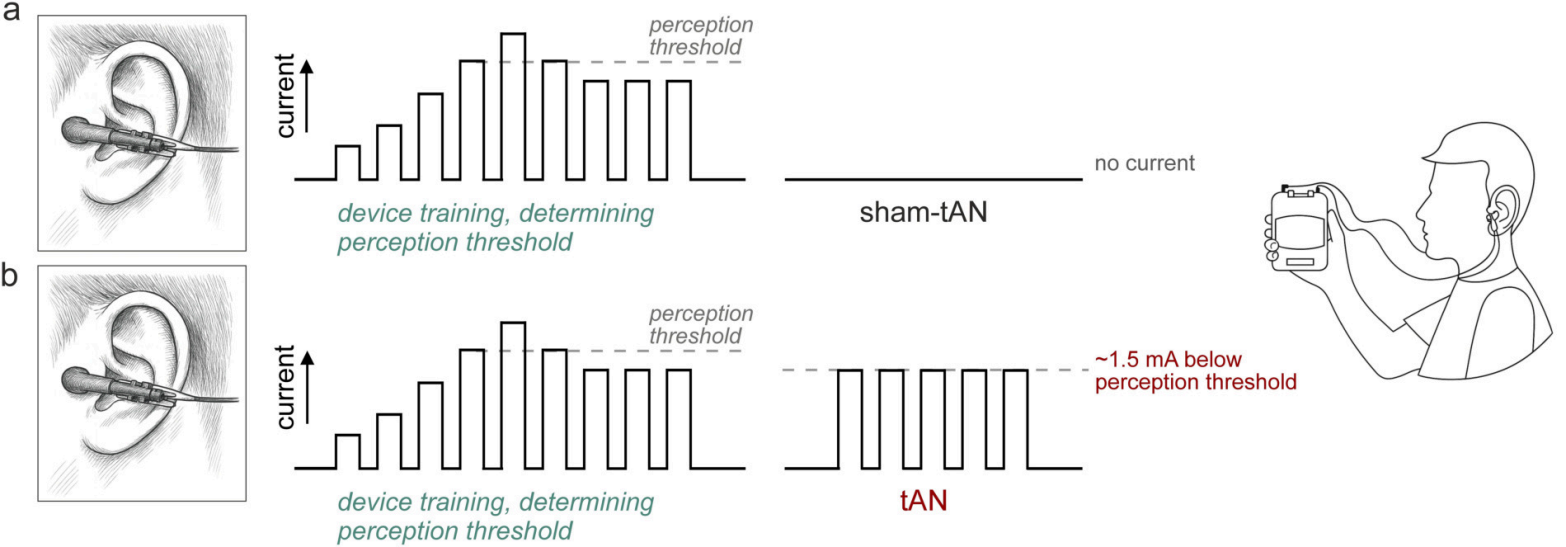
AffeX-CT device settings. The individual sensitivities of the auricular tragi regions to tAN stimulation were determined during the randomisation visit using a dedicated training device. During device training, participants were instructed to place the electrode clips on the left and right tragi. The current amplitude was gradually increased by the investigator conducting the training, starting from 0.1 mA, until the participant felt a tingling sensation. The current was then reduced to set the level of stimulation at ∼1.5 mA below this threshold. Once this threshold current was determined, participants were issued their personal device units, identical to the training device but with concealed controls. The stimulation current was set to 0 mA (**a**; sham-tAN) or ∼1.5 mA below the individual perception threshold (**b**; tAN), with 200 *μ*s pulses generated at a frequency of 30 Hz.

During device training, participants were instructed to place the electrode clips (with electrode surfaces made of electrically conductive rubber) on the left and right tragi (Figure 1). The current amplitude was gradually increased by the trainer, starting from 0.1 mA, until the participant felt a tingling sensation, after which it was reduced to set the level of stimulation at ∼1.5 mA below this threshold (Figure 1). Once the threshold current was determined, participants were issued their individual units, which were identical to the training device but with concealed controls. Stimulation current was set to 0 mA (sham-tAN) or ∼1.5 mA below the individual perception threshold (tAN), with 200 *μ*s pulses generated at a frequency of 30 Hz.

Participants were instructed to use the device independently at home, ideally setting aside 30 min in the evening when relaxed and not engaging in strenuous activity. Light activities such as reading or watching TV were recommended during stimulation. Before using the device, participants cleaned the outer ear. The device's two ear clip leads (left and right) were moistened with a wet tissue at the contact points and attached to the left and right tragi. The device was then activated to stimulate the auricular branch of the vagus nerve.

For participants in the sham-tAN arm, the same training procedure was followed. However, the sham AffeX-CT device was programmed to deliver no stimulation current to the tragus. Otherwise, the sham device was identical in all other aspects. The participants, clinical research team, and data analysis team were all blinded to the intervention.

### Procedure

**Figure 2:**
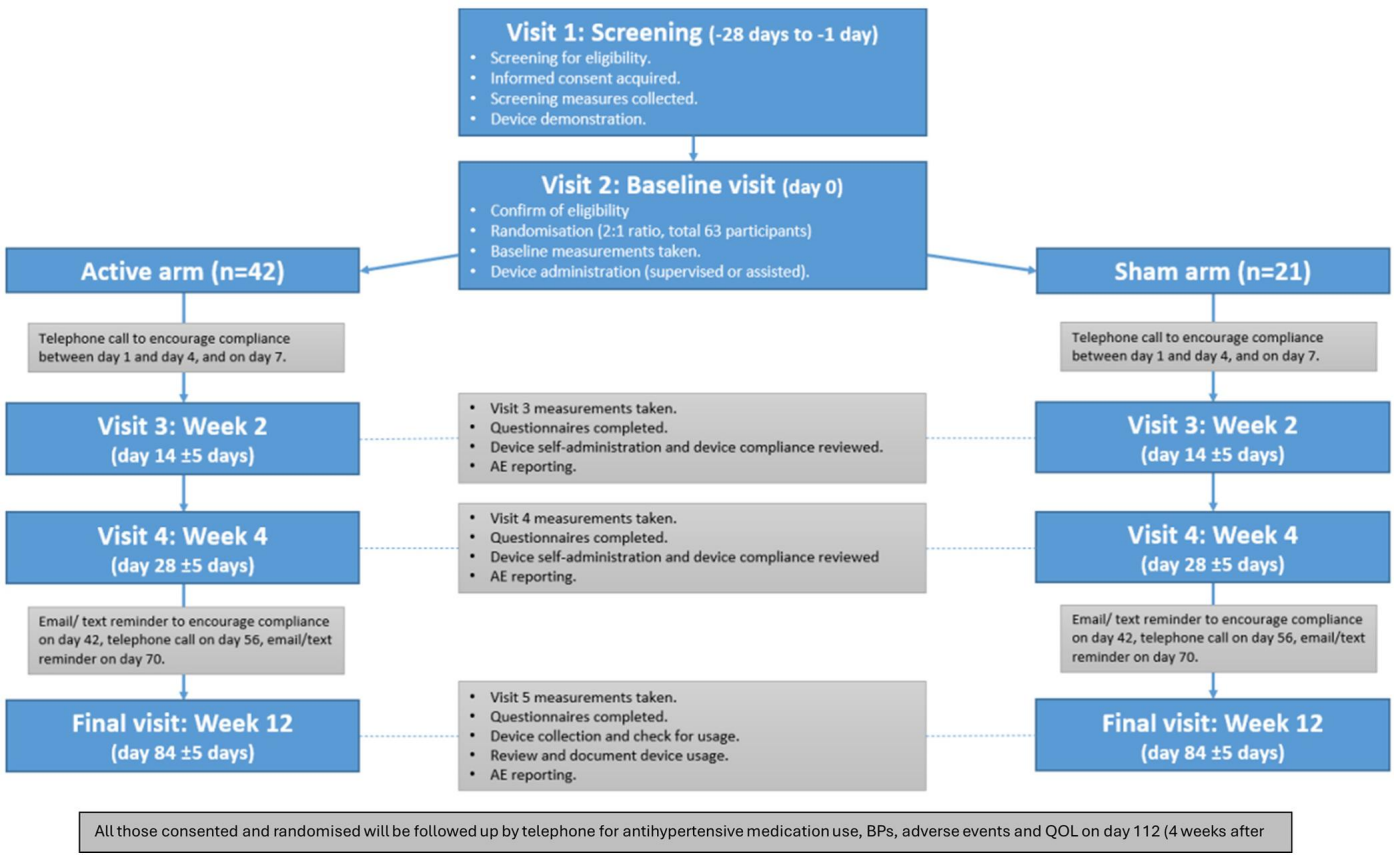
Flowchart for SCRATCH-HTN main trial.

Trial participants were required to attend five visits spanning 12 weeks/84 days: visit 1 for the screening; visit 2 for baseline and randomisation at Day 0; visit 3 at Day 14; visit 4 at Day 28; and visit 5 for the end of treatment at Day 84 (see Figure 2 for Flowchart). Participants also received several telephone calls: one between days 1 and 3, one on Day 7, one at Day 56, and one at Day 112 (post-trial follow-up). Participants received text and/or email reminders at Day 42 and Day 70. The expected total duration of participation was 16 weeks. No changes in antihypertensive medications were permitted during the trial (between randomisation on Day 0/visit 2 and end of treatment on Day 84/visit 5). Table 2 summarises the assessments and data collected at each visit with a full detailed schedule provided in Supplementary Table 4.

**Table 2:** Summary schedule of assessments and timeline of data collected.

Schedule	Visit 1 (Screening Visit)	Visit 2 (Baseline/Randomisation Visit)	Phone Call 1	Phone Call 2	Visit 3	Visit 4	Text/Email Reminder1	Phone Call 3	Text/Email Reminder2	Visit 5 (End of treatment visit)	Phone Call 4 Follow-up
Timeline (weeks/days)	n/a	Week 0 Day 0	Day 1-4	Week 1 Day 7	Week 2 Day 14	Week 4 Day 28	Week 6 Day 42	Week 8 Day 56	Week 10 Day 70	Week 12 Day 84	Week 16 Day 112
Visit window	28 days (−1 day)	n/a	n/a	±3 days	±5 days	±5 days	±3 days	±3 days	±3 days	±5 days	±3 days
Informed consent medical, demographic and social history Inclusion & Exclusion criteria reviewed	X										
Vital signs	X^a^	X			X	X				X	
Weight & BMI	X^b^	X								X	
Review of concomitant medication(s) and AE	X	X	X	X	X	X		X		X	X
24-hour ABPM	X	X^c^				X				X	
Office BP	X	X^d^			X	X				X^d^	
24-hour Holter ECG		X				X				X	
6-minute walk test (6 MWT)		X			X	X				X	
Echocardiogram		X								X	
Electrocardiogram (ECG)	X										
Blood test^e^	X	X				X				X	
Urine sample^f^		X				X				X	
Device procedure and logbook reviewed		X	X	X	X	X	X	X	X	X	
Extent of adherence scales questionnaire		X			X					X	
Insomnia severity index (ISI) Questionnaire		X				X				X	
Blinding and AffeX-CT device usability questionnaire						X				X	
EQ-5D QoL questionnaire^g^ cognitive assessment		X			X	X				X	

^a^ X Height and waist circumference collected at visit 1.

^b^ Vital signs Pulse rate, respiratory rate, temperature, and oxygen saturation assessments.

^c^ X Screening 24-h ABPM will be used at baseline, only if within screening period and no subsequent treatment changes have been made. Otherwise, 24-h ABPM must be repeated at baseline (randomisation) visit.

^d^ X Central BPs will also be taken at visits 2 and 5.

^e^ Blood Test Full blood count (FBC), lipid profile, glucose (fasting), HbA1c, fructosamine, U&Es, Serum pregnancy for female participants on screening and randomisation visits. Blood samples for storage and later evaluations (plasma and serum) will also be taken at visits 2, 4, and 5.

^f^ Urine Sample Urinary albumin creatinine ratio and urinary antihypertensive drug screen. Urine pregnancy test for women <55 years age at visits 1 and 2.

g X EQ-5D QoL Questionnaire also completed at week 16, Phone Call 4 Follow-up.

A maximum of 28 days was allowed between screening and randomisation. At the screening visit (visit 1), all participants provided informed signed consent to enrol into the study, along with demographic information, medical and social history, and current concomitant medications. Vital signs, height and waist circumference, weight and BMI, ECG, 24-h ABPM, and office BP assessments were collected. Blood samples were taken from all participants, and for female participants <55 years of age, serum and urine pregnancy testing was completed. Inclusion and exclusion criteria were evaluated during the screening period. At the chief investigator's (CI) discretion, the screening visit could be repeated once. Only after all screening assessments were completed, and once the participant was eligible and consented to the trial, were they invited to attend the baseline/randomisation visit (visit 2).

At baseline and randomisation (visit 2, Day 0), participants were trained to use the device and provided with their personal AffeX-CT devices (including a user manual, see Supplementary Material 2—User Guide), device logbook (to record the date, time, and duration of each stimulation session and the presence of any adverse effects), and participant ID card. Patients also underwent the following assessments: vital signs, weight and BMI, 24-h ABPM, office BP, central BP, 24-h Holter ECG, 6-min walk test (6 MWT), echocardiogram, blood tests (for pathology assessment and for storage and later evaluations), and urine tests (for urinary albumin–creatinine ratio and urinary antihypertensive drug screening to objectively assess adherence to medications).The trial participants were trained on using the device, prescribed an individualised stimulation intensity as described earlier, issued their assigned randomised device, and asked to perform the first self-stimulation under the direct supervision of the clinical research staff. Participants had their safety BPs recorded after 30 min of their first self-stimulation. Participant also completed three questionnaires [Extent of Adherence scale (39), Insomnia Severity Index (ISI) (40), and EuroQol Five-Dimensional scale for Quality of Life (EQ-5D QoL)] (41) and a cognitive assessment (PEBL 2.0) (42).

Participants self-administered the treatment using the device for 14 consecutive days, 30 min each evening. During this period, participants received two phone calls as reminders to apply treatment daily for the duration of the initial course of treatment and complete device procedure and device logbook entries. Concomitant medication and adverse events (AEs) were simultaneously reviewed.

After 14 days, visit 3 was conducted where medications, logbooks, and AEs were reviewed. The participants also underwent office BP measurement, vital sign assessment, and 6 MWT and completed the cognitive assessment, adherence, and QoL questionnaires. Participants were then asked to self-administer therapy once a week for a further 10 weeks.

Visit 4 took place on Day 28. At this visit, vital signs, 24-h ABPM, office BP, 24-h Holter ECG, and 6 MWT were assessed, and a review of concomitant medication(s), use of the device, logbook, and AEs was completed. Participants had their blood and urine samples collected for safety and medication adherence assessments and also completed cognitive assessment and questionnaires on device usability, QoL, insomnia, and blinding. Email/text reminders to complete the device procedure and logbook entry were sent at Day 42. A phone call was conducted at Day 56 reminding participants to continue using the device and completing logbook entries, while concomitant medication(s) and AEs were reviewed. Another email/text reminder was sent on Day 70.

The end-of-treatment visit (visit 5) took place at the end of week 12 (Day 84). Participants completed the following assessments: vital signs, weight and BMI, 24-h ABPM, office BP, central BP, 24-h Holter ECG, 6 MWT, echocardiogram, and safety blood and urine samples. Concomitant medication(s), device logbooks, and AEs were reviewed, and the device was returned. Participants then completed all trial questionnaires and the final cognitive assessment.

After the trial, follow-up was conducted at week 16 (Day 112) with a phone call to review concomitant medication(s), AEs, and the QoL remotely.

In addition, an extra visit/clinical assessment was offered as per each participant's clinical requirement, particularly for those with either high (SBP > 180 mmHg) or low (SBP < 100 mmHg) BP, symptomatic postural hypotension, or other clinical concerns.

### Outcomes

The primary endpoint was the change in average daytime ambulatory SBP from baseline to the end of treatment (3 months). Both assessments of average daytime ambulatory SBP were calculated using ABPM recordings between 7 a.m. and 11 p.m. on the day of the assessment. Secondary endpoints, detailed in Supplementary Table 5, included changes in 24-h ambulatory and office SBP and DBP, ambulatory HR, BP variability, HR variability, quality of life, and sleep quality from baseline to end of treatment.

### Exploratory endpoints and sub-studies

The trial also collected data throughout the study (Supplementary Table 4) to assess various exploratory endpoints (see Supplementary Table 6 for details.) These included feasibility endpoints to assess the safety and acceptability of the device for patients, echocardiographic markers, and central BP measurements at baseline and end of treatment to explore the potential broader cardiovascular effects of tAN. Plasma and serum samples were also stored at −80°C from blood collected at baseline, week 4, and week 12 visits for all patients for subsequent analysis of proteomics, inflammatory markers, and metabolomics.

For a subgroup of consented patients (*n* = 22), we completed a sub-study of several detailed physiological tests, commonly known as Autonomic Target-Organs Neurophysiological tests (ATONT), at baseline and week 12 (end of treatment), to explore the potential mechanism underlying the effects of tAN. ATONT takes approximately 90 min to complete and includes continuous measurements of heart rate, beat-to-beat BP, tissue oxygenation, respiratory rate, and ECG monitoring. Participants also completed simple physiological manoeuvres: standing from lying position, hand-grip, sit up, Valsalva manoeuvre, and carotid massage.

### Sample size

The trial was powered for the primary endpoint (change in daytime ambulatory SBP between baseline and the end of the treatment at 12 weeks) in the active treatment arm. This was done using a paired *t*-test approach.

Using a conservative assumption of a mean change in SBP of 5.5 mmHg with a standard deviation of 11 mmHg, based on the existing data for hypertensive patients, 34 participants were required to give 80% power to detect such a change at the two-sided alpha level of 0.05. After inflation for a potential 10% drop-out and a further 10% non-compliance, we required 42 subjects in the intervention (tAN) arm.

The study also recruited participants to be randomised to the sham-tAN arm to compare changes in SBP in those undergoing treatment to consider potential Hawthorne and placebo effects. In order to collect more data regarding safety in the active treatment group, the trial recruited double the number of subjects for the active treatment than for the sham treatment—a randomisation ratio of 2:1. Therefore, the sample size for the sham-tAN treatment was 21, with a total sample size across both arms of 63 subjects.

The sample size of 63 subjects (42 tAN, 21 sham), using a 2:1 randomisation ratio, provided 80% power to detect a between-arm difference of 8.4 mmHg in daytime SBP assuming no attrition, or 9.3 mmHg assuming a 20% attrition (10% dropout plus 10% non-compliance).

### Statistical methods

Baseline demographic and physiological characteristics will be presented separately by trial group. The main analyses of the primary and secondary endpoints will be conducted on an intention-to-treat population (all randomised participants with available data irrespective of their compliance with the device) and on a per-protocol population (all participants with treatment compliance on 80% or more days during the trial). Any participants who required a change in antihypertensive medication during the treatment period for safety/ethical reasons or otherwise will be included in the intention-to-treat analyses but not in the per-protocol analyses.

The primary endpoint, change in daytime ambulatory SBP, will be calculated for each participant by subtracting the end-of-treatment measurement (at 3 months) from the baseline measurement. The mean change in daytime SBP within each group will be presented with 95% confidence intervals (CIs) within the active treatment arm. The crude difference in the mean change in daytime SBP between treatment arms will be calculated with a 95% CI, and a two-sample *t*-test with equal variances will be used to test the null hypothesis of no difference in the change in SBP between groups.

Adjusted analyses will be conducted using linear regression, comparing change in SBP between treatment groups while adjusting for baseline daytime ABPM SBP (ANCOVA), age, sex, and BMI. The estimated adjusted difference in change in SBP will be presented, along with 95% CIs and *p*-values. The primary endpoint will also be analysed according to subgroups defined by (i) BMI at baseline (<30 kg/m^2^ vs. ≥30 kg/m^2^), (ii) diabetes status at baseline, (iii) age at baseline (<65 years vs. ≥65 years), and (iv) mean ABPM daytime SBP at baseline (<160 mmHg vs. ≥160 mmHg); for each of these four risk factors, a test for interaction will be conducted between groups. Moreover, data from the urinary antihypertensive drug screening samples collected at visits 2, 4, and 5, providing objective longitudinal data on antihypertensive medication adherence for each participant from the point of randomisation to end of treatment, will be used to adjust the analysis of the primary outcome for partial or non-compliance to medications.

All secondary endpoints that are continuous will be analysed in the same way as for the primary endpoint. Binary endpoints will be displayed as the whole number of subjects who meet that endpoint at the end of the study alongside the total number of subjects in that group. A percentage of subjects meeting that endpoint for each group will also be recorded. For binary endpoints, the crude and adjusted odds ratios (ORs) will be estimated.

All feasibility endpoints for this phase 2a study will be presented descriptively or analysed qualitatively.

We will also summarise AEs using counts and percentages and present these overall for the study and by treatment arm. The number of subjects with AEs of mild/moderate/severe intensity will be shown overall and by treatment arm using the maximum severity experienced by each participant. The total number of AEs for each treatment, allowing multiple events per participant, will also be presented.

Serious AEs (SAEs; both non-fatal and fatal) will be listed separately along with details of the treatment and whether the event is unexpected or thought to be related to the treatment.

No interim analyses of any endpoint were planned. The full statistical analysis plan is provided in Supplementary Material 3, and the full clinical investigational plan is provided in Supplementary Material 4.

## Discussion

The SCRATCH-HTN trial is a double-blind, sham-controlled study evaluating the safety and acceptability of tAN using the AffeX-CT device and a novel stimulation algorithm for the first time in human subjects. The trial employs an improved protocol involving bilateral tragus stimulation. In addition to assessing safety, the study investigates the potential efficacy of tAN in reducing BP in patients with uncontrolled hypertension despite ongoing treatment. The findings are expected to pave the way for further evaluation of this innovative technology and establish it as a viable therapeutic option. Through the number of secondary and exploratory endpoints and the sub-study, the SCRATCH-HTN trial is designed to be hypothesis-generating. By examining the effects of tAN on sleep quality, exercise tolerance, heart rate variability, central BP, cardiac structure, and autonomic function, the study aims to provide mechanistic insights and data on related potential therapeutic benefits that may inform future studies. If effective, or if tAN demonstrates efficacy within the constraints of statistical power considerations, tAN could represent a transformative approach to hypertension management, fostering further research into this device and its applications in the field.

The study was carefully designed to mitigate known risks associated with device-based and non-pharmacological interventions in hypertension trials. The trial participants were required to have uncontrolled BP despite receiving medication, who were asked to avoid any changes in their medication. Although there is an increased risk of cardiovascular sequelae from uncontrolled hypertension, the trial duration was just 12 weeks, a shorter period than that which patients invariably wait between community and secondary care visits following dose escalation. Those patients at higher risk with recent complications of hypertension such as stroke, myocardial infarction, or decompensated heart failure that require strict control of BP were excluded from the trial at screening. Moreover, we carefully monitored the patients during the trial period and if their BPs were at levels that could potentially damage target organs acutely, for example, mean average office SBP > 180 mmHg or DBP > 120 mmHg, we allowed the introduction of another medication or escalation of existing antihypertensive treatment as per the current guidelines and their physician’s advice.

Participants in device trials are often particularly motivated by the prospect of innovative treatments, which can introduce biases. To minimise this, all enrolled participants were already on established antihypertensive therapy at baseline. At both screening and randomisation, they were explicitly informed that their medication regimen must remain unchanged throughout the study. To reinforce adherence and detect any compliance issues, multiple checks were implemented: verbal confirmation, self-reported data via participant device logbooks, and regular urinary screening for antihypertensive agents. These measures aimed to support protocol fidelity and capture any non-adherence.

Regarding potential risks directly related to the use of the AffeX-CT device, there are no known serious risks of applying this form of autonomic neuromodulation. In a study of six healthy volunteers, no adverse effects of tAN administered using the AffeX-CT device on heart rate, BP, or ECG parameters (QT interval) were observed during 30 min of stimulation and for 60 min after the stimulation. No side effects and/or AEs were reported by the patients recruited in the proof-of-concept study (19 participants). Moreover, systematic reviews and meta-analyses show that tAN/tVNS is a safe and well-tolerated treatment (43, 44). Incidence of AEs, in general, are low, with one meta-analysis calculating 12.84/100,000 person-min-days of stimulation (44). Another meta-analysis reported that in 1,322 human subjects, the most common side effects were local skin irritation from electrode placement (240 subjects, 18.2%), headache (47 subjects, 3.6%), and nasopharyngitis (23 subjects, 1.7%) (43). In the SCRATCH-HTN study, the clinical investigation site team ensured that preventative measures and proper technique for device stimulation sessions (such as correct skin preparation before stimulation) were reinforced throughout the study to reduce the risk of adverse events such as local skin reactions. Adverse events were reviewed during every visit and phone call. In cases of persistent or more serious device-associated local skin reactions, investigators could review the stimulation current, advise on the application of mild emollients after stimulation sessions, or pause stimulation altogether if required to ensure the safety of the participants remained the priority. Other anticipated side effects with a frequency of <1% included light-headedness, fatigue/tiredness, mood changes, neck pain, tooth pain, pain/local skin irritation due to attachment of the device ear clips, tingling sensation due to the use of the device, and increased frequency of ventricular extrasystoles. Most of these side effects are transient and temporary in nature, and are likely to reduce in intensity and frequency over regular usage. Another challenge of device trials is to maintain blinding amongst participants—particularly those in the active arm. Theoretically, participants can self-adjust the stimulation level and may infer their group allocation. Our training protocol for the participants reduced this risk, as did the constant reminders throughout the study period. A recent study using a similar device in healthy volunteers (45) reported minimal risk of unblinding across two arms of the study. Moreover, in our study, we collected specific data to assess whether perceptions of treatment assignment differed between groups, and we will perform sensitivity analyses, if required.

If the findings of the proof-of-concept study are confirmed (reduction in SBP by >10 mmHg, lasting for >1 month after the discontinuation of therapy), tAN will prove to be more efficacious than renal denervation or most single antihypertensive medications. The implication of lowering BP by 5–10 mmHg is significant. The largest and most detailed individual patient-level meta-analysis of data obtained from 348,854 participants across 48 randomised clinical trials (evaluating the effects of BP-lowering treatments on the risk of major cardiovascular events and death in patients with and without cardiovascular disease) demonstrated that over an average of 4 years of follow-up, each 5 mmHg reduction in SBP lowered the relative risk of major cardiovascular events by ∼10%. The risks of stroke, ischaemic heart disease, heart failure, and death from cardiovascular disease are reduced by 13%, 8%, 13%, and 5%, respectively, with each 5 mmHg reduction in SBP (46). For 21 participants randomised to receive the sham-tAN treatment, we anticipate participation in the trial to be beneficial not only due to the Hawthorne effect but also because of the overall care and comprehensive cardiovascular assessment provided during the trial.

Overall, this study is expected to provide evidence not only on the safety and acceptability of tAN but also on the physiological and mechanistic understanding of whether non-invasive autonomic neuromodulation via auricular stimulation can improve BP control, overall wellbeing, exercise tolerance, and other outcomes outlined in the primary and secondary objectives. Amongst these, evidence supporting a potential effect on BP control would be particularly significant, potentially paving the way for more in-depth investigations and offering a novel therapeutic option for people with uncontrolled hypertension.

## Dissemination

The trial was registered on ClinicalTrials.gov (NCT05179343) and ISRCTN (14509154) and received Clinical Trial No Objection (CI/2021/0069/GB) from the Medicines and Healthcare products Regulatory Agency (MHRA), a favourable opinion from the West of Scotland NHS Research Ethics Committee (21/WS/0157), and Health Research Authority Approval. After the publication of the main results and planned sub-studies, the data, analysis code, and fully disclosed results (including those of the secondary, exploratory, and sub-study outcomes) may be shared upon reasonable request to the chief investigator to promote reproducibility and future collaborations.

Recruitment for the SCRATCH-HTN study was completed in May 2025. Final participant follow-ups were due to be completed in September 2025, with the analysis and subsequent presentation of results, in peer-reviewed scientific journals and at scientific conferences, anticipated in early 2026.

After the publication of the main results and planned sub-studies, the data, analysis code, and fully disclosed results (including those of the secondary, exploratory, and sub-study outcomes) may be shared upon reasonable request to the chief investigator to promote reproducibility and future collaborations. Recruitment for the SCRATCH-HTN study was completed in May 2025. Final participant follow-ups were due to be completed in September 2025, with the analysis and subsequent presentation of results, in peer-reviewed scientific journals and at scientific conferences, anticipated in early 2026.

## SCRATCH HTN Study investigators

### SCRATCH-HTN investigators

**William Harvey Research Centre, Queen Mary University of London, and Barts Health NHS Trust:** Ajay Gupta, Jane Field, David Collier, James Steckelmacher, Olivier Zongo, Mital Patel, Patrizia Ebano, Manish Saxena, Abubaker Eltayeb, Annastazia Learoyd, Georgia Mannion-Krase, Clovel David, Mussadiq Shah, Peter Julu, and Amrita Ahluwalia.

**Imaging team**: Jing Deng, James Malcolmson, and Sanjeev Bhattacharyya.

**PIC sites investigators:** Royal Free London NHS Foundation Trust: Alfredo Petrosino (PI), Stephen Walsh,* and Manoj Makharia; University College London Hospitals NHS Foundation Trust: Marc George (PI) and Mollie Little; St George's University Hospitals NHS Foundation Trust: Teck Khong (PI); Homerton University Hospital NHS Foundation Trust: Louise Abram (PI); Broomfield Hospital, Mid and South Essex Hospitals NHS Foundation Trust: Tehreem Butt (PI), Rachael Arnold, and Caroline Mitchell; and Imperial College Healthcare NHS Trust: Neil Poulter (PI) and Farhat Ghafoor.

* Posthumous

### SCRATCH HTN Trial Committees

**Trial Management Committee (TMG):** Ajay Gupta (chair), David Collier, James Steckelmacher, Jane Field, Olivier Zongo, Mital Patel, George Collett, Annastazia Learoyd, Abubaker Eltayeb, Manish Saxena, Georgia Mannion-Krase, and Patrizia Ebano.

**Trial Steering Committee (TSC):** Peter Sever (chair), Ian Wilkinson, Rasha Al-Lamee, Ken Peters, Peter Davison, Ajay Gupta, David Collier, Annastazia Learoyd, Alexander Gourine, Everard Mascarenhas, Lucia Bianchi, Stuart Haylock, and Jane Field.

**Data Safety Monitoring Committee (DSMC):** Neil Chapman (chair), Vikas Kapil, Tim Collier, Ajay Gupta, Kamran Khan, Annastazia Learoyd, Jane Field, and Raj Mehta.

**Research Steering Group (RSG):** Ajay Gupta (chair), Paul Robinson, Gareth Ackland, Alexander Gourine, Everard Mascarenhas, Rayan Altayeb, Mamta Bajre, and Jane Field.

## References

[1] WHO. Global Report on Hypertension: The Race Against a Silent Killer. Geneva: World Health Organization (2023).

[2] GBD 2021 Risk Factors Collaborators. Global burden and strength of evidence for 88 risk factors in 204 countries and 811 subnational locations, 1990–2021: a systematic analysis for the Global Burden of Disease Study 2021. Lancet. (2024) 403:2162–203. 10.1016/S0140-6736(24)00933-4 38762324 PMC11120204

[3] GBD 2021 Causes of Death Collaborators. Global burden of 288 causes of death and life expectancy decomposition in 204 countries and territories and 811 subnational locations, 1990-2021: a systematic analysis for the Global Burden of Disease Study 2021. Lancet. (2024) 403:2100–32. 10.1016/S0140-6736(24)00367-2 38582094 PMC11126520

[4] GBD 2021 Risk Factors Collaborators. Global burden of 87 risk factors in 204 countries and territories, 1990-2019: a systematic analysis for the Global Burden of Disease Study 2019. Lancet. (2020) 396:1223–49. 10.1016/S0140-6736(20)30752-2 33069327 PMC7566194

[5] GBD Chronic Kidney Disease Collaboration. Global, regional, and national burden of chronic kidney disease, 1990-2017: a systematic analysis for the Global Burden of Disease Study 2017. Lancet. (2020) 395:709–33. 10.1016/S0140-6736(20)30045-3 32061315 PMC7049905

[6] GBD 2019 Stroke Collaborators. Global, regional, and national burden of stroke and its risk factors, 1990-2019: a systematic analysis for the Global Burden of Disease Study 2019. Lancet Neurol. (2021) 20:795–820. 10.1016/S1474-4422(21)00252-0 34487721 PMC8443449

[7] Mensah GA Fuster V Murray CJL Roth GA, Global Burden of Cardiovascular Diseases and Risks Collaborators. Global Burden of Cardiovascular Diseases and Risks, 1990-2022. J Am Coll Cardiol. (2023) 82:2350–473. 10.1016/j.jacc.2023.11.007 38092509 PMC7615984

[8] Official Statistics, National statistics, Survey. Health Survey for England, 2021, Part 2. Leeds: NHS Digital (2023).

[9] Graham C Steckelmacher J Prashar J Ahmed A Capel M Poulter NR Trends in hypertension prevalence, control and antihypertensive use in England over the last 2 decades: insights from annual, nationwide Health Surveys for England from 2003 to 2021. BMJ Med. (2025) 4:e001556. 10.1136/bmjmed-2025-001556 41333849 PMC12666190

[10] Public Health England. Tackling High Blood Pressure: From Evidence into Action. London: Public Health England (2014).

[11] Lee EKP Poon P Yip BHK Bo Y Zhu M-T Yu C-P Global burden, regional differences, trends, and health consequences of medication nonadherence for hypertension during 2010 to 2020: a meta-analysis involving 27 million patients. J Am Heart Assoc. (2022) 11:e026582. 10.1161/JAHA.122.026582 36056737 PMC9496433

[12] Abegaz TM Shehab A Gebreyohannes EA Bhagavathula AS Elnour AA. Nonadherence to antihypertensive drugs: a systematic review and meta-analysis. Medicine (Baltimore). (2017) 96:e5641. 10.1097/MD.0000000000005641 28121920 PMC5287944

[13] Bourque G Ilin JV Ruzicka M Hundemer GL Shorr R Hiremath S. Nonadherence is common in patients with apparent resistant hypertension: a systematic review and meta-analysis. Am J Hypertens. (2023) 36:394–403. 10.1093/ajh/hpad013 36715101

[14] Dahal K Khan M Siddiqui N Mina G Katikaneni P Modi K Renal denervation in the management of hypertension: a meta-analysis of sham-controlled trials. Cardiovasc Revasc Med. (2020) 21:532–7. 10.1016/j.carrev.2019.07.012 31420197

[15] Gupta A Prince M Bob-Manuel T Jenkins JS. Renal denervation: alternative treatment options for hypertension? Prog Cardiovasc Dis. (2020) 63:51–7. 10.1016/j.pcad.2019.12.007 31884099

[16] Kandzari DE Böhm M Mahfoud F Townsend RR Weber MA Pocock S Effect of renal denervation on blood pressure in the presence of antihypertensive drugs: 6-month efficacy and safety results from the SPYRAL HTN-ON MED proof-of-concept randomised trial. Lancet. (2018) 391:2346–55. 10.1016/S0140-6736(18)30951-6 29803589

[17] Mufarrih SH Qureshi NQ Khan MS Kazimuddin M Secemsky E Bloch MJ Randomized trials of renal denervation for uncontrolled hypertension: an updated meta-analysis. J Am Heart Assoc. (2024) 13:e034910. 10.1161/JAHA.124.034910 39140334 PMC11963938

[18] Farmer AD Strzelczyk A Finisguerra A Gourine AV Gharabaghi A Hasan A International consensus based review and recommendations for minimum reporting standards in research on transcutaneous vagus nerve stimulation (version 2020). Front Hum Neurosci. (2021) 14:568051. 10.3389/fnhum.2020.568051 33854421 PMC8040977

[19] Clancy JA Mary DA Witte KK Greenwood JP Deuchars SA Deuchars J. Non-invasive vagus nerve stimulation in healthy humans reduces sympathetic nerve activity. Brain Stimul. (2014) 7:871–7. 10.1016/j.brs.2014.07.031 25164906

[20] Yuan H Silberstein SD. Vagus nerve and vagus nerve stimulation, a comprehensive review: part II. Headache. (2016) 56:259–66. 10.1111/head.12650 26381725

[21] Yuan H Silberstein SD. Vagus nerve and vagus nerve stimulation, a comprehensive review: part III. Headache. (2016) 56:479–90. 10.1111/head.12649 26364805

[22] Yuan H Silberstein SD. Vagus nerve and vagus nerve stimulation, a comprehensive review: part I. Headache. (2016) 56:71–8. 10.1111/head.12647 26364692

[23] Bretherton B Atkinson L Murray A Clancy J Deuchars S Deuchars J. Effects of transcutaneous vagus nerve stimulation in individuals aged 55 years or above: potential benefits of daily stimulation. Aging. (2019) 11:4836–57. 10.18632/aging.102074 31358702 PMC6682519

[24] Frangos E Ellrich J Komisaruk BR. Non-invasive access to the vagus nerve central projections via electrical stimulation of the external ear: fMRI evidence in humans. Brain Stimul. (2015) 8:624–36. 10.1016/j.brs.2014.11.018 25573069 PMC4458242

[25] Garcia RG Lin RL Lee J Kim J Barbieri R Sclocco R Modulation of brainstem activity and connectivity by respiratory-gated auricular vagal afferent nerve stimulation in migraine patients. Pain. (2017) 158:1461–72. 10.1097/j.pain.0000000000000930 28541256 PMC5517046

[26] Kraus T Kiess O Hösl K Terekhin P Kornhuber J Forster C. CNS BOLD fMRI effects of sham-controlled transcutaneous electrical nerve stimulation in the left outer auditory canal—a pilot study. Brain Stimul. (2013) 6:798–804. 10.1016/j.brs.2013.01.011 23453934

[27] Antonino D Teixeira AL Maia-Lopes PM Souza MC Sabino-Carvalho JL Murray AR Non-invasive vagus nerve stimulation acutely improves spontaneous cardiac baroreflex sensitivity in healthy young men: a randomized placebo-controlled trial. Brain Stimul. (2017) 10:875–81. 10.1016/j.brs.2017.05.006 28566194

[28] De Couck M Cserjesi R Caers R Zijlstra WP Widjaja D Wolf N Effects of short and prolonged transcutaneous vagus nerve stimulation on heart rate variability in healthy subjects. Auton Neurosci. (2017) 203:88–96. 10.1016/j.autneu.2016.11.003 28017263

[29] Yang H Shi W Fan J Wang X Song Y Lian Y Transcutaneous auricular vagus nerve stimulation (ta-VNS) for treatment of drug-resistant epilepsy: a randomized, double-blind clinical trial. Neurotherapeutics. (2023) 20:870–80. 10.1007/s13311-023-01353-9 36995682 PMC10275831

[30] He W Jing X Wang X Rong P Li L Shi H Transcutaneous auricular vagus nerve stimulation as a complementary therapy for pediatric epilepsy: a pilot trial. Epilepsy Behav. (2013) 28:343–6. 10.1016/j.yebeh.2013.02.001 23820114

[31] Zamotrinsky A Afanasiev S Karpov RS Cherniavsky A. Effects of electrostimulation of the vagus afferent endings in patients with coronary artery disease. Coron Artery Dis. (1997) 8:551–7. 9431484

[32] Stavrakis S Stoner JA Humphrey MB Morris L Filiberti A Reynolds JC TREAT AF (transcutaneous electrical vagus nerve stimulation to suppress atrial fibrillation): a randomized clinical trial. JACC Clin Electrophysiol. (2020) 6:282–91. 10.1016/j.jacep.2019.11.008 32192678 PMC7100921

[33] Stavrakis S Humphrey MB Scherlag BJ Hu Y Jackman WM Nakagawa H Low-level transcutaneous electrical vagus nerve stimulation suppresses atrial fibrillation. J Am Coll Cardiol. (2015) 65:867–75. 10.1016/j.jacc.2014.12.026 25744003 PMC4352201

[34] Mbikyo MB Wang A Ma Q Miao L Cui N Yang Y Low-level tragus stimulation attenuates blood pressure in young individuals with hypertension: results from a small-scale single-blind controlled randomized clinical trial. J Am Heart Assoc. (2024) 13:e032269. 10.1161/jaha.123.032269 39291497 PMC11681467

[35] Stavrakis S Elkholey K Morris L Niewiadomska M Asad ZUA Humphrey MB. Neuromodulation of inflammation to treat heart failure with preserved ejection fraction: a pilot randomized clinical trial. J Am Heart Assoc. (2022) 11:e023582. 10.1161/jaha.121.023582 35023349 PMC9238491

[36] Hays SA Rennaker RL Kilgard MP. “Targeting plasticity with vagus nerve stimulation to treat neurological disease”. In: Merzenich MM Nahum M Van Vleet TM, editors. Changing Brains—Applying Brain Plasticity to Advance and Recover Human Ability. Amsterdam: Elsevier (2013). p. 275–99. 10.1016/B978-0-444-63327-9.00010-2 PMC4615598 24309259

[37] Kwong PWH Ng GYF Chung RCK Ng SSM. Bilateral transcutaneous electrical nerve stimulation improves lower-limb motor function in subjects with chronic stroke: a randomized controlled trial. J Am Heart Assoc. (2018) 7:e007341. 10.1161/jaha.117.007341 29437598 PMC5850185

[38] Kuznetsova OM Tymofyeyev Y. Preserving the allocation ratio at every allocation with biased coin randomization and minimization in studies with unequal allocation. Stat Med. (2012) 31:701–23. 10.1002/sim.4447 22161821

[39] Voils CI Maciejewski ML Hoyle RH Reeve BB Gallagher P Bryson CL Initial validation of a self-report measure of the extent of and reasons for medication nonadherence. Med Care. (2012) 50:1013–9. 10.1097/MLR.0b013e318269e121 22922431 PMC3494794

[40] Bastien CH Vallieres A Morin CM. Validation of the Insomnia Severity Index as an outcome measure for insomnia research. Sleep Med. (2001) 2:297–307. 10.1016/s1389-9457(00)00065-4 11438246

[41] Balestroni G Bertolotti G. EuroQol-5D (EQ-5D): an instrument for measuring quality of life. Monaldi Arch Chest Dis. (2012) 78:155–9. 10.4081/monaldi.2012.121 23614330

[42] Mueller ST Piper BJ. The psychology experiment building language (PEBL) and PEBL test battery. J Neurosci Methods. (2014) 222:250–9. 10.1016/j.jneumeth.2013.10.024 24269254 PMC3897935

[43] Redgrave J Day D Leung H Laud PJ Ali A Lindert R Safety and tolerability of transcutaneous vagus Nerve stimulation in humans; a systematic review. Brain Stimul. (2018) 11:1225–38. 10.1016/j.brs.2018.08.010 30217648

[44] Kim AY Marduy A de Melo PS Gianlorenco AC Kim CK Choi H Safety of transcutaneous auricular vagus nerve stimulation (taVNS): a systematic review and meta-analysis. Sci Rep. (2022) 12:22055. 10.1038/s41598-022-25864-1 36543841 PMC9772204

[45] Ackland GL Patel ABU Miller S Gutierrez del Arroyo A Thirugnanasambanthar J Ravindran JI Non-invasive vagus nerve stimulation and exercise capacity in healthy volunteers: a randomized trial. Eur Heart J. (2025) 46:1634–44. 10.1093/eurheartj/ehaf037 39969124 PMC7617618

[46] Blood Pressure Lowering Treatment Trialists' Collaboration. Pharmacological blood pressure lowering for primary and secondary prevention of cardiovascular disease across different levels of blood pressure: an individual participant-level data meta-analysis. Lancet. (2021) 397:1625–36. 10.1016/S0140-6736(21)00590-0 33933205 PMC8102467

